# Retrospective clinical study of an implant with a sandblasted, large-grit, acid-etched surface and internal connection: analysis of short-term success rate and marginal bone loss

**DOI:** 10.1186/s40902-016-0089-6

**Published:** 2016-11-05

**Authors:** Jae-Wang Lee, Jun Hyeong An, Sang-Hoon Park, Jong-Hyon Chong, Gwang-Seok Kim, JeongJoon Han, Seunggon Jung, Min-suk Kook, Hee-Kyun Oh, Sun-Youl Ryu, Hong-Ju Park

**Affiliations:** Department of Oral and Maxillofacial Surgery, School of Dentistry, Chonnam National University, 77 Yongbong-ro, Buk-gu, Gwangju, Republic of Korea

**Keywords:** Sandblasted, large-grit, acid-etched surface, Success rate, Marginal bone loss, Implant

## Abstract

**Background:**

The purpose of this retrospective study was to evaluate the clinical utility of an implant with a sandblasted, large-grit, acid-etched (SLA) surface and internal connection.

**Methods:**

Six patients who received dental implants in the Department of Oral and Maxillofacial Surgery, Chonnam National University Dental Hospital, were analyzed by factors influencing the success rate and marginal bone loss. Factors included patient’s age, sex, implant installation site, whether bone graft was done, type of bone graft materials, approaching method if sinus lift was done, and the size of the fixture. In addition, the marginal bone loss was analyzed by using a radiograph.

**Results:**

All implants were successful, and the cumulative survival rate was 100 %. Average marginal bone loss of 6 months after the installation was 0.52 mm and 20 months after the functional loading was 1.06 mm. Total marginal bone resorption was 1.58 mm on average. There was no statistically significant difference in mesial and distal marginal bone loss.

**Conclusions:**

The short-term clinical success rate of the implant with an SLA surface and internal connection was satisfactory. Moreover, the marginal bone loss was also consistent with the implant success criteria.

## Background

Restoring the occlusal function with a dental implant is now popular and generally recognized as highly predictable. Compared to other treatment methods using removable dentures, it results in better occlusal function and shows less discomfort in denture-supporting areas. Moreover, patients show more satisfaction [1–6].

However, the lack of primary and secondary stability due to bone resorption leads to implant failure [7, 8]. Successful implant requires osseointegration, which can be achieved by early implant fixation and bone healing. According to Branemark, osseointegration is a direct contact between the implant surface and the bone that can be seen under the magnification of an optical microscope [9–11]. Albrektsson defined the clinical meaning of osseointegration as a firm fixation caused by a heterogeneous material and the maintenance of the bone without any symptoms during functional occlusion [9, 12].

Osseointegration is necessary for implant stability and proper functional load [9, 13]. Primary implant stability, in particular, is affected not only by the bone quality and volume but also by design, surface treatment, length, diameter, and other factors that an implant has [7, 14–16]. Over 95–96 % implant success rate has been achieved worldwide, and there are many suggestions for implant surface treatment to increase biocompatibility and bone-forming ability of the host [9, 17–22]. There have been many studies showing implant success. It is noted that there are many factors affecting the long-term success of an implant. Marginal bone height is important in determining the functional and esthetic success [1, 23]. It is also compulsory to keep the proper height of the marginal bone for long-term success.

Sandblasted, large-grit, acid-etched (SLA) implant that underwent acid etching treatment after body cut using high-pressure large metal grain particles shows better early osseointegration than differently surface-treated implants. Such fact has been demonstrated in animal studies [24–28]. However, studies on the clinical usefulness of the SLA implant are not sufficient at this point.

The main purpose of this study was to evaluate the clinical usefulness of SLA implants. This study retrospectively analyzed clinical and radiographic data after implant fixation and application of functional load. Sex, age, and systemic disease of patients and fixation location, diameter, and length of the implants were also checked in order to analyze the effect of such factors on the marginal bone loss.

## Methods

### Patients and materials

This study retrospectively analyzed radiographs of six patients who had implant surgery from July 2012 to June 2014, using 32 implants with the Shinhung® Luna system. Of six patients, there were four men and two women between the ages of 23 and 65 years and an average age of 53.5 years. Conditions of patients with systemic disease (one with hypertension, one with hypertension and diabetes, and one with hepatitis B) were well controlled.

The Shinhung® Luna system fixture used in this study is an internal submerged type, and its tapered body can obtain firm and stable early fixation in any bone quality. Three helical cutting edges allow self-tapping that can ease implant installation and minimize the resistance of the bone. Gradually enlarging thread exerts pressure on the bone and makes it favorable for stable insertion and by decreasing bone damage. Its upper open-type part enables less resistant installation without any additional drilling. Double thread with 35° spiral helix empowers quick and firm installation. An SLA surface showing an average roughness of over Ra 2.5 μm improved bone healing period and cell response by more than 20 %, respectively. In addition, outstanding early fixation was achieved in soft bones.

There were 17 implants with 11.5-mm, 9 with 10.0-mm, 5 with 13.0-mm, and 1 with 8.5-mm length. There were 20 implants with 5.0-mm, 8 with 4.0-mm, and 4 with 4.5-mm diameter (Table 1).

**Table 1:** Number of implants placed according to implant length and diameter

	8.5 mm	10.0 mm	11.5 mm	13.0 mm	Total
4.0 mm	0	3	3	2	8
4.5 mm	0	1	3	0	4
5.0 mm	1	5	11	3	20
Total	1	9	17	5	32

Among 32 implants, 21 were installed in the maxilla and 11 were installed in the mandible. Detailed information of the implant locations is described in Table 2.

**Table 2:** Number of implants placed according to location in the arch

Location	Maxilla	Mandible	Total
Premolar	8	1	9
Molar	13	10	23
Total	21	11	32

Several kinds of bone graft materials were used for maxillary sinus floor elevation. The mandibular ramus was used for autogenous bone graft and Bio-Oss® (Geistlich, Wolhusen, Switzerland) for heterogeneous bone graft.

### Surgical procedures

Local anesthesia was administered prior to implant installation. Implant was installed following the full-thickness flap elevation according to the recommendation of the manufacturer. Crestal or lateral approach was applied when the maxillary sinus elevation was needed. Maxillary sinus elevation was performed in 18 out of 21 cases of the maxilla. Sixteen out of 18 implants were performed with bone graft and the other two without it. In the case of bone graft, the autogenous bone was used solely in 13 cases. A mixture of autogenous and heterogeneous bones was used in three cases (Table 3).

**Table 3:** Type of sinus bone graft materials

Type of bone graft material	Number of cases (%)
Mandibular ramus bone	13 (81.3)
Mandibular ramus bone + Bio-Oss®	3 (18.7)
Total	16 (100)

### Assessments

#### Analysis of medical records

Referring to the medical records of patients, their sex, age, and systemic disease, the location of the implant installation, and its length and diameter were investigated. According to such data, the difference in the duration of the implant success and their effects towards marginal bone resorption were evaluated.

The criteria of success are as follows [3, 24, 29, 30]:

- The absence of mobility assessed manually and by a manual torque test
- The absence of peri-implant radiolucency
- The absence of continuous pain or suppuration around the implant
- The absence of deep (>5 mm) pockets adjacent to the implant
- Bone loss <4 mm

The criteria of survival are as follows [31, 32]:

- The absence of pain, foreign body sensation, and dysesthesia
- The absence of recurrent peri-implant infection with suppuration
- The absence of mobility
- The absence of continuous radiolucency around the implant

#### Analysis of radiographs

Radiographs were taken after the implant installation, 6 months after the installation, and after the functional loading. To calculate the marginal bone loss, distance from the top level of the implant platform to the marginal bone contact level on the uppermost part of the implant was measured. In addition, mesial and distal parts of the implant were measured each time. The marginal bone resorption levels were measured right after the implant installation, 6 months after the installation, and after the functional loading.

**Fig. 1:**
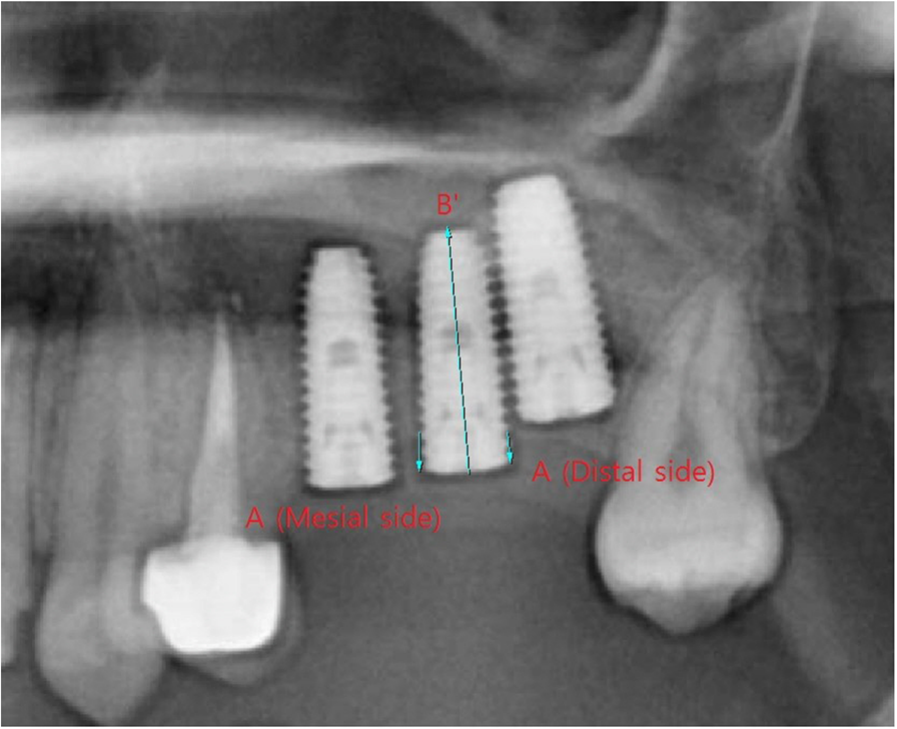
Measurement of the marginal bone loss

The length of the implant fixture was set as a standard in radiographic correction. The distance from the top level of the implant platform to the marginal bone contact level on the uppermost part of the implant (*A*) and the length of the implant fixture (*B*) were measured and corrected as follows to get the actual distance from the top level of the implant platform to the marginal bone contact level on the uppermost part of the implant (*X*) (Fig. 1).

$$ X= AB\ /\ B' $$

where *A* is the distance from the top level of the implant platform to the marginal bone contact level on the uppermost part of the implant (measurement value), *B* is the length of the implant fixture (actual value), and *B*’ is the length of the implant fixture (measurement value).

The periodic marginal bone resorption was quantified by subtracting previously measured marginal bone length from the one measured each time.

### Statistical analysis

In order to compare the marginal bone resorption at different parts of the fixture, paired sample *t* test was performed. Independent sample *t* test was applied for measuring the difference in marginal bone resorption according to patients’ sex, the location of the installation site, and systemic disease. For statistical analysis, SPSS (version 17.0 for Windows, SPSS Inc., Chicago, IL, USA) was used.

## Results

### Marginal bone loss around implants

Marginal bone loss of 32 implants was measured periodically. The average marginal bone loss at 6 months after the installation was 0.52 mm and at 20 months after the functional loading was 1.06 mm. Total marginal bone resorption was 1.58 mm on average. There was no statistically significant difference in the mesial and distal marginal bone loss (Table 4).

**Table 4:** Marginal bone loss around implants according to the observation period

	Marginal bone resorption (mm) (mean ± SD)		
	Mesial	Distal	*t* test *P* value
Sur-6 months	0.59 ± 0.77	0.46 ± 0.47	.394
Prosthesis	1.19 ± 1.38	0.92 ± 0.94	.193
Total	1.78 ± 1.79	1.38 ± 1.29	.173

*Sur-6 months* the period from surgery to 6 months, *Prosthesis* the period from functional loading to 20 months, *SD* standard deviation

### Marginal bone loss between men and women

Marginal bone loss was compared between sexes. From the installation to after the functional loading, the average marginal bone loss was 1.02 mm in men and 2.52 mm in women. The marginal bone loss showed significant differences in the distal area at 6 months after the installation and both mesial and distal areas at 20 months after the functional loading (Table 5).

**Table 5:** Comparison of marginal bone loss between men and women

	Site	Sex	Implant observed	Bone resorption (mm) (mean ± SD)	*t* test *P* value
Sur-6 months	Mesial	Male	20	0.56 ± 0.86	.809
		Female	12	0.63 ± 0.65	
	Distal	Male	20	0.23 ± 0.21	.002
		Female	12	0.84 ± 0.53	
Prosthesis	Mesial	Male	20	0.77 ± 1.33	.021
		Female	12	1.90 ± 1.19	
	Distal	Male	20	0.48 ± 0.49	.003
		Female	12	1.67 ± 1.06	
Total	Mesial	Male	20	1.33 ± 1.81	.064
		Female	12	2.54 ± 1.55	
	Distal	Male	20	0.71 ± 0.52	.001
		Female	12	2.51 ± 1.41	

*Sur-6 months* the period from surgery to 6 months, *Prosthesis* the period from functional loading to 20 months, *SD* standard deviation

### Marginal bone loss according to the region in the arch

The difference in marginal bone loss between the premolar and molar regions was evaluated periodically. The average loss was 2.01 mm in the premolar and 1.42 mm in the molar region at the point of installation until an average of 20 months after the functional loading. However, the differences did not suggest any statistical significance (Table 6).

**Table 6:** Comparison of marginal bone loss according to the regions in the arch

	Site	Location	Implant observed	Bone resorption (mm) (mean ± SD)	*t* test *P* value
Sur-6 months	Mesial	Premolar	9	1.10 ± 1.27	.133
		Molar	23	0.39 ± 0.33	
	Distal	Premolar	9	0.47 ± 0.50	.957
		Molar	23	0.46 ± 0.46	
Prosthesis	Mesial	Premolar	9	1.39 ± 1.23	.626
		Molar	23	1.12 ± 1.45	
	Distal	Premolar	9	1.06 ± 1.13	.608
		Molar	23	0.87 ± 0.88	
Total	Mesial	Premolar	9	2.50 ± 2.17	.166
		Molar	23	1.51 ± 1.59	
	Distal	Premolar	9	1.53 ± 1.57	.692
		Molar	23	1.33 ± 1.19	

*Sur-6 months* the period from surgery to 6 months, *Prosthesis* the period from functional loading to 20 months, *SD* standard deviation

The marginal bone loss between the maxilla and mandible was evaluated. The maxillary loss was 1.72 mm and mandibular loss was 1.32 mm on average from the point of installation to an average period of 20 months after the functional loading. Despite the greater loss in the maxilla, no significant difference was found (Table 7).

**Table 7:** Comparison of marginal bone loss between the maxilla and mandible

	Site	Location	Implant observed	Bone resorption (mm) (mean ± SD)	*t* test *P* value
Sur-6 months	Mesial	Maxilla	21	0.73 ± 0.91	.070
		Mandible	11	0.32 ± 0.28	
	Distal	Maxilla	21	0.41 ± 0.37	.511
		Mandible	11	0.55 ± 0.62	
Prosthesis	Mesial	Maxilla	21	1.38 ± 1.46	.306
		Mandible	11	0.84 ± 1.20	
	Distal	Maxilla	21	0.93 ± 0.94	.985
		Mandible	11	0.92 ± 0.99	
Total	Mesial	Maxilla	21	2.11 ± 1.92	.161
		Mandible	11	1.16 ± 1.38	
	Distal	Maxilla	21	1.34 ± 1.20	.789
		Mandible	11	1.47 ± 1.49	

*Sur-6 month* the period from surgery to 6 months, *Prosthesis* the period from functional loading to 20 months, *SD* standard deviation

### Marginal bone loss according to systemic disease

From the point of installation to the functional loading, marginal bone loss of patients with the systemic disease was 1.07 mm on average while that of healthy patients was 2.34 mm. Until 6 months after the installation and until an average period of 20 months after the functional loading, particularly, the distal area showed a significant difference (Table 8).

**Table 8:** Comparison of marginal bone loss according to systemic disease

	Site	Disease	Implant observed	Bone resorption (mm) (mean ± SD)	*t* test *P* value
Sur-6 months	Mesial	+	19	0.59 ± 0.87	.977
		−	13	0.59 ± 0.65	
	Distal	+	19	0.24 ± 0.22	.004
		−	13	0.78 ± 0.55	
Prosthesis	Mesial	+	19	0.80 ± 1.36	.051
		−	13	1.76 ± 1.25	
	Distal	+	19	0.50 ± 0.49	.006
		−	13	1.54 ± 1.11	
Total	Mesial	+	19	1.40 ± 1.84	.142
		−	13	2.35 ± 1.63	
	Distal	+	19	0.74 ± 0.52	.002
		−	13	2.33 ± 1.50	

*Sur-6 month* the period from surgery to 6 months, *Prosthesis* the period from functional loading to 20 months, *SD* standard deviation

### Cumulative survival rate and success rate of implants

Among the 32 implants, none was removed during the study period. The cumulative survival rate was 100 %. At the final progress review of the observations, two implants showed more than baseline marginal bone loss. The success rate was 93.8 %.

## Discussion

In this retrospective study, all implants were successful and the cumulative survival rate was 100 %. The cumulative survival rates of implants with an internal connection in various studies were 95.3 % at 5 years [33, 34], 96.8 % at 8 years [3, 33], and 96.2 % at 10 years [33, 35]. The observation period of this study was more than 1 year after loading. However, it is shorter than the period of the previous study. Considering the suggestion of several authors, the evaluation of the marginal bone resorption after a period of more than a year after loading could be used as one of the indicators to predict the long-term prognosis of the implant because most implant failures occur within 1 year after loading. The implant failure mainly occurred within 1 year after loading, and the failure rate dramatically reduced 1 year after loading [36]. The causes of marginal bone loss within 1 year after loading were surgical trauma, excessive force placed on the crestal bone, traumatic occlusion, unfavorable jaw relationship, cantilever extensions, physiological residual ridge resorption, and inflammation [2, 37].

In this study, the radiographs were taken to evaluate and compare the marginal bone resorption in accordance with the observation period. The average marginal bone loss at 6 months after the implant installation was 0.52 mm and after an average of 20 months after the functional loading was 1.06 mm. The total marginal bone loss was 1.58 mm on average. A bone loss of up to 1.5 mm in the first year after loading is considered acceptable as part of biological remodeling according to the criteria by Albrektsson [29]. According to the success criteria of Albrektsson and others [24], the absence of mobility and less than 4-mm bone loss was considered as a successful implant. It means that the Shinhung® Luna system fixture could be successfully used clinically. Its tapered body can obtain firm and stable early fixation in any bone quality. Gradually enlarging thread exerts pressure on the bone in stages and makes it favorable by decreasing bone damage and for stable installation.

Recently, SLA implants have been shown to have even better early osseointegration. In animal studies, the mean bone–implant contact was 30–40 % for titanium plasma-sprayed (TPS) implants but 50–60 % for SLA implants [24, 25]. The implant failure was higher in the resorbable blast media (RBM) group compared to the SLA group, and these results were significant [38].

In this study, the marginal bone loss was greater in women than in men. There were significant differences in the distal area after 6 months and both mesial and distal areas after the functional loading.

According to Wyatt and Zarb [37], no sex difference was seen in the mean annual bone loss after the first year of loading. In other studies, there were no significant differences between the sexes for the success rate of the implant [1, 39, 40].

In the present study, there were no significant differences between the jaws or the regions in the arch for marginal bone loss. Similarly, Naert et al. [40] observed that neither the jaw site nor the implant position (anterior–posterior) had any significant effect on the outcome of a marginal bone loss. Wyatt and Zarb [37] also mentioned that statistical analyses were unable to show any relationships between bone loss and bone quantity and quality, implant location within and between the jaws, or implant length after the first year of loading. When evaluating the measured bone loss, a higher mean amount of bone loss was observed in the maxilla as compared with the mandible; however, this was not statistically significant [1, 41].

In this study, the marginal bone loss was less in patients who had systemic diseases than healthy patients. There were significant differences in 6 months after the installation and after the functional loading at the distal area. Similarly, Mombelli and Cionca [42] observed that failure rates were not different between 98 systemically healthy subjects and 109 patients with a history of other systemic diseases. Moy et al. [1, 39] also mentioned that sex, hypertension, coronary artery disease, pulmonary disease, steroid therapy, chemotherapy, and not being on hormone replacement therapy for postmenopausal women were not associated with a significant increase in implant failure.

## Conclusions

The short-term clinical success rate of the implant with an SLA surface and internal connection was satisfactory. Moreover, the marginal bone loss was also consistent with the implant success criteria. The results of this retrospective study demonstrated that the implant with an SLA surface and internal connection can be restored with a high predictability of success. Since the overall failure rate is very low, more studies with higher subject numbers and longer follow-up are required.
